# Fluoride Induces Endoplasmic Reticulum Stress and Inhibits Protein Synthesis and Secretion

**DOI:** 10.1289/ehp.11375

**Published:** 2008-05-21

**Authors:** Ramaswamy Sharma, Masahiro Tsuchiya, John D. Bartlett

**Affiliations:** 1 Department of Cytokine Biology, Forsyth Institute, Boston, Massachusetts, USA; 2 Department of Developmental Biology, Harvard School of Dental Medicine, Boston, Massachusetts, USA; 3 Division of Aging and Geriatric Dentistry, Graduate School of Dentistry, Tohoku University, Sendai, Japan

**Keywords:** ameloblasts, eIF2α, enamel, endoplasmic reticulum, ER stress, fluoride, LS8 cells, SEAP, UPR

## Abstract

**Background:**

Exposure to excessive amounts of fluoride (F^−^) causes dental fluorosis in susceptible individuals; however, the mechanism of F^−^-induced toxicity is unclear. Previously, we have shown that high-dose F^−^ activates the unfolded protein response (UPR) in ameloblasts that are responsible for dental enamel formation. The UPR is a signaling pathway responsible for either alleviating endoplasmic reticulum (ER) stress or for inducing apoptosis of the stressed cells.

**Objectives:**

In this study we determined if low-dose F^−^ causes ER stress and activates the UPR, and we also determined whether F^−^ interferes with the secretion of proteins from the ER.

**Methods:**

We stably transfected the ameloblast-derived LS8 cell line with secreted alkaline phosphatase (SEAP) and determined activity and localization of SEAP and F^−^-mediated induction of UPR proteins. Also, incisors from mice given drinking water containing various concentrations of F^−^ were examined for eucaryotic initiation factor-2, subunit alpha (eIF2α) phosphorylation.

**Results:**

We found that F^−^ decreases the extracellular secretion of SEAP in a linear, dose-dependent manner. We also found a corresponding increase in the intracellular accumulation of SEAP after exposure to F^−^. These changes are associated with the induction of UPR proteins such as the molecular chaperone BiP and phosphorylation of the UPR sensor PKR-like ER kinase, and its substrate, eIF2α. Importantly, F^−^-induced phosphorylation of eIF2αwas confirmed *in vivo*.

**Conclusions:**

These data suggest that F^−^ initiates an ER stress response in ameloblasts that interferes with protein synthesis and secretion. Consequently, ameloblast function during enamel development may be impaired, and this may culminate in dental fluorosis.

Fluoride is anticariogenic and is recommended by the U.S. Public Health Service for addition to drinking water at a concentration of 0.7–1.2 ppm, such that an average of 1 mg F^−^ is consumed per day [Centers for Disease Control and Prevention (CDC) 1995]. However, higher levels of F^−^ exposure can result in dental fluorosis, which is manifested as mottled, discolored, porous enamel that is susceptible to decay (DenBesten 1999). High F^−^ doses can cause skeletal fluorosis that may result in bone fracture (Boivin et al. 1989). High F^−^ doses may also cause renal toxicity (Zager and Iwata 1997), epithelial lung cell toxicity (Thrane et al. 2001), and reproductive defects (Ghosh et al. 2002). Among these, attention has been focused on the role of F^−^ in dental fluorosis, because the most apparent effects of excess F^−^ ingestion in an individual are white spots (mild fluorosis) or dark stains (moderate to severe fluorosis) on the teeth.

Ameloblasts are epithelial cells that are responsible for enamel formation. The three major stages of the ameloblast life cycle, namely, secretory, transition, and maturation, correspond to distinct steps in enamel development. During the secretory stage, the ameloblasts are tall and columnar, and they secrete large amounts of proteins that form a matrix within which thin enamel ribbons of hydroxyapatite crystallize. Once the enamel ribbons attain their full length, ameloblasts enter the transition stage, when they decrease in height and experience a reduction in Golgi complex and rough endoplasmic reticulum (ER). During the maturation stage, the ameloblasts secrete KLK4 (Simmer et al. 1998) to help degrade the enamel proteins, which are then resorbed from the maturing enamel. It is during the maturation stage that the enamel ribbons grow in width and thickness to form mature hardened enamel. Normal enamel is composed of about 96% mineral and 4% organic content (Robinson et al. 1988). Excess F^−^ ingestion during tooth formation causes an increase in the protein content and a decrease in overall mineral content of the enamel (Robinson et al. 2004; Robinson and Kirkham 1990; Wright et al. 1996). F^−^ ions have been suggested to adversely affect the precipitation of hydroxyapatite that forms the mineralized enamel (Aoba and Fejerskov 2002). However, two observations suggest that F^−^-mediated toxicity also involves genetic responses. First, different inbred strains of mice with similar overall levels of F^−^ in their enamel differ in their susceptibilities to fluorosis (Everett et al. 2002). Second, no correlation was found between the concentration of F^−^ in enamel and the severity of dental fluorosis (Vieira et al. 2004). These results clearly suggest a genetic basis for susceptibility to fluorosis (Yan et al. 2007). We have previously shown that F^−^induces ER stress in ameloblasts, thereby compromising their function during enamel formation (Kubota et al. 2005).

Proteins to be secreted are translocated into the ER for posttranslational modification, folding, and assembly. The ER is a quality control organelle in which individual proteins must adopt a stable conformation; unfolded or misfolded proteins are prevented from traversing the secretory pathway (Hammond and Helenius 1995). Factors that compromise ER homeostasis initiate an ER-to-nucleus signaling pathway, termed the unfolded protein response (UPR). Activation of the UPR serves three major functions: *a*) it results in transcriptional up-regulation of molecular chaperones such as BiP/GRP78 that help augment the folding capacity of the ER; *b*) it transiently attenuates protein translation via phosphorylation of the translation initiation factor, eucaryotic initiation factor-2, subunit alpha (eIF2α), thereby allowing cells to cope with the existing protein load; and *c*) it activates components of the ER-associated degradative pathway (ERAD) to degrade the accumulated mis-folded proteins. If these pathways succeed in alleviating cell stress, the cell survives; if not, the cell undergoes apoptosis via caspase activation. Indeed, ER stress is associated with several diverse diseases, including diabetes, neurodegenerative disorders (Gow and Sharma 2003), arsenite exposure (Lu et al. 2001), and heavy metal–induced toxicity (Hiramatsu et al. 2007).

Recently, the reporter construct secreted alkaline phosphatase (SEAP) (Berger et al. 1988) was used to detect and quantify ER stress in real time (Hiramatsu et al. 2006b). SEAP traverses the secretory pathway (Lara-Lemus et al. 2006), and its activity can be detected at very low levels (0.2 pg/mL). Because SEAP is a secreted protein, medium supernatant can be assayed for SEAP activity in a real-time fashion. Most importantly, transfection of cells with SEAP does not, by itself, cause ER stress (Hiramatsu et al. 2006b). SEAP secretion is decreased only by ER stress-inducing agents such as tunicamycin that blocks N-linked glycosylation or thapsigargin that functions as an inhibitor of Ca^2 +^ ATPase (Hiramatsu et al. 2007). Cytokines that do not cause ER stress, such as tumor necrosis factor-α,transforming growth factor-β, or interleukin-1β, do not decrease SEAP activity (Hiramatsu et al. 2006b). Thus, SEAP is specifically sensitive to ER stress-inducing agents. SEAP has been used to detect ER stress induced by heavy metals such as nickel, cadmium, and cobalt in cell lines as well as in mice (Hiramatsu et al. 2007).

In this study, we demonstrate that F^−^ concentrations as low as 2.4 ppm can induce ER stress in LS8 cells, which results in the inhibition of protein secretion; we also identify PKR-like ER kinase (PERK)-mediated phosphorylation of eIF2α as a signaling pathway responsible for F^−^-mediated inhibition of protein synthesis.

## Materials and Methods

### Production of stable transfectant

pSEAP2-Control (Great EscAPe SEAP Reporter System 3) and pTK-Hygromycin (both from Clontech, Mountain View, CA, USA) were cotransfected into LS-8 cells using Lipofectamine-LTX reagent and Lipofectamine Plus Reagent (both from Invitrogen, Carlsbad, CA, USA). After 24 hr, cells were washed and grown in alpha-MEM medium (Invitrogen) containing 750 μg/mL Hygromycin B (Invitrogen). Positive clones, isolated using cloning rings, were assayed for SEAP activity using the Great EscAPe SEAP Chemiluminescence Kit 2.0 (Clontech). Clone 10 showed the highest activity and was used for all experiments. For negative control, LS-8 cells were stably transfected with pTK-Hygromycin and pSEAP2-Basic (Great EscAPe SEAP Reporter System 3; Clontech), that lacks the SV40 early promoter and enhancer sequences present in pSEAP2-Control. Cells were maintained in alpha-MEM containing 10% fetal bovine serum and 750 μg/mL Hygromycin B during all experiments.

### SEAP activity assay

We measured SEAP activity using the Great EscAPe SEAP Chemiluminescence Kit 2.0 according to the manufacturer’s instructions. Briefly, 25 μL cell supernatant was mixed with 75 μL of 1×dilution buffer in 96-well plates and incubated at 65°C for 30 min. Plates were chilled for 3 min, and 100 μL SEAP substrate was added. After incubating at room temperature for 30 min, samples were measured for chemiluminescence on a Victor 1420 multilabel counter (PerkinElmer, Waltham, MA, USA); data are expressed in relative light units (RLU). All experiments were performed in triplicate and were repeated three times.

### Immunoblotting

Cells were grown in 100-mm plates and treated with varying doses of sodium F^−^ for either 6 or 24 hr. Supernatant was collected for assessing the quantity and activity of SEAP released into the medium. To detect UPR proteins and intra-cellular SEAP, cells were washed twice with phosphate-buffered saline (PBS, pH 7.4) and lysed with Complete Lysis-M reagent (Roche Diagnostics, Mannheim, Germany) containing protease and phosphatase inhibitors (Roche Diagnostics). We determined the protein concentration using the BCA assay kit (Pierce, Rockford, IL, USA). A total of 10–30 μg protein was loaded per lane onto 4–20% polyacrylamide gels (Bio-Rad Laboratories, Hercules, CA, USA). Proteins were transferred onto nitrocellulose membranes (Schleicher and Schuell, Whatman, Germany), blocked with 5% nonfat dried milk, and probed with primary antibodies in blocking solution overnight at room temperature. Blots were washed with PBS containing 0.1% Tween-20 (PBST) and incubated with secondary antibodies conjugated to horseradish peroxidase for 1 hr at room temperature. After washing with PBST, bands were developed with ECL Advance Western Blotting kit (GE Healthcare, Piscataway, NJ, USA). We used the following primary antibodies: goat anti-PLAP (placental alkaline phosphatase; 1:1000) and goat anti-BiP (immunoglobulin heavy chain binding protein; 1:1000) from Santa Cruz Biotechnology Inc. (Santa Cruz, CA, USA); rabbit anti-eIF2α (1:1000, [pS52]; BioSource, Camarillo, CA, USA), and mouse anti-actin (1:500; Sigma, St. Louis, MO, USA). Secondary antibodies were purchased from Southern Biotech (Birmingham, AL, USA).

### Immunocytochemistry and immunohisto-chemistry

LS8-SEAP cells were grown on four-chamber tissue culture-treated glass slides (BD Biosciences, Bedford, MA, USA) and treated with 0.125 mM (2.4 ppm) sodium fluoride (NaF) for 24 hr. Cells were fixed with 2% paraformaldehyde for 30 min and permeabilized with 0.2% Triton X-100 for 30 min. After washing with PBS (pH 7.4), cells were blocked with 10% goat serum in PBS for 1 hr and treated with primary antibody (Rb monoclonal anti-phospho-PERK, 1:100; Cell Signaling Technology, Danvers, MA, USA) in blocking solution overnight. After washing with PBS, secondary antibody (Alexa 488-conjugated goat anti-rabbit IgG, 1:2000; Invitrogen) in blocking solution was added for 1 hr. Antifade containing 2,4-diamino-diphenyl indole (DAPI) was then added, and slides were cover-slipped and sealed before imaging under a Leica DM RX 2 microscope (Leica Microsystems, Bannockburn, IL, USA) running Axiovision version 5.0 (Carl Zeiss Microimaging, Thornwood, NY, USA)

Immunohistochemistry was performed as described previously by Kubota et al. (2005). Briefly, 6-week-old C57BL/6J mice were given water containing different concentrations of F^−^ (0, 25, 50, and 100 ppm) *ad libitum* for 3–4 weeks. Mice were sacrificed, and incisors were formalin-fixed, paraffin-embedded, and sectioned. Sections were blocked with 10% goat serum in PBS and incubated overnight with rabbit anti-phospho-eIF2α (1:200, [pS^52^]; BioSource) followed by incubation in peroxidase-conjugated antibody (Vectastain Elite Reagent, Vector Labs, Burlingame CA, USA) and in Sigma Fast 3,3’-diaminobenzidine substrate (Sigma). Sections were examined by light microscopy and photographed. All animals were treated humanely and with regard for alleviation of suffering according to institutional animal care and use committee guidelines.

### Cell proliferation assay

LS8-SEAP cells were plated at a density of 2,500 cells in 96-well plates. After 18 hr, medium was changed and cells were incubated for either 6 or 24 hr in 100 μL medium containing varying doses of F^−^. For measuring cell proliferation, 10 μL WST-1 (Roche Diagnostics) was added, and the resulting absorbance was measured after 3 hr at 440 nm on an EL800 Universal Microplate Reader (Biotek Instruments, Inc, Winooski, VT, USA). All experiments were performed in triplicate and repeated three times.

### Statistics

We performed one-way analysis of variance with Bonferroni’s posttest using GraphPad Prism, version 5.00 for Windows (GraphPad Software, San Diego CA, USA).

## Results

### Dose-dependent decrease in SEAP secretion

**Figure 1:**
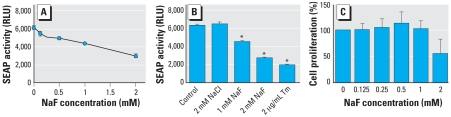
Effect of F^−^ on SEAP secretion in LS8-SEAP cells treated with F^−^ for 6 hr. (*A*) Linear dose–dependent decrease in SEAP activity (*p* < 0.001) after NaF treatment. (*B*) Decrease in SEAP activity after treatment with F^−^ or the ER stress-inducing agent tunicamycin (Tm); NaCl has no effect. (*C*) Relationship between the 6 hr F^−^-mediated decrease in SEAP activity and cell proliferation. All experiments were performed in triplicate and performed three times *p < 0.0001.

The ameloblast-derived LS8 cells were stably transfected with SEAP expression plasmid to generate cells (LS8-SEAP) that constitutively secrete SEAP. LS8-SEAP cells were treated with medium containing 0.0, 0.125, 0.25, 0.5, and 1.0 mM F^−^ (corresponding to 0, 2.4, 4.8, 9.5, and 19 ppm F^−^, respectively). Aliquots of cell supernatant were removed at 6 hr and assayed for SEAP activity. As a positive control, cells were treated with the N-linked glycosylation inhibitor tunicamycin. As shown in Figure 1A, SEAP activity decreased in a linear, dose-dependent manner with increasing concentrations of F^−^. A significant decrease was observed within 6 hr with the lowest F^−^ dose tested (0.125 mM F^−^; *p* < 0.01) and became highly significant (*p* < 0.001) for each of the F^−^ concentrations > 0.125 mM. Cell proliferation, quantified by the WST-1 assay, showed no significant difference at 6 hr (Figure 1C). Similarly, the assay for lactate dehydrogenase, a cytoplasmic enzyme that is released into the medium on cell death, showed no significant F^−^-induced cell cytotoxicity at 6 hr (data not shown). Thus, the observed decrease in SEAP activity did not correlate to cell proliferation or cell death. Treatment of cells with sodium chloride did not have any significant effect on SEAP activity, suggesting that the F^−^ ion was responsible for the observed effects (Figure 1B). Tunicamycin also decreased SEAP activity, confirming previous reports that SEAP can be used to detect ER stress (Hiramatsu et al. 2006b). Therefore, F^−^ treatment attenuated the secretion of SEAP from LS8-SEAP cells.

### Lack of direct inhibition of SEAP activity

**Figure 2:**
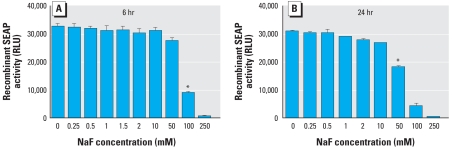
Effect of low-dose F^−^ on SEAP activity. Recombinant SEAP collected from untreated LS8-SEAP cells was directly treated with NaF for 6 hr (*A*) or 24 hr (*B*), and SEAP activity was measured. A significant decrease in SEAP activity was observed only with doses > 50 mM NaF, suggesting that F^−^ does not directly inhibit SEAP activity. Data are averages of three separate experiments *p < 0.001.

Flouride, at a high concentration of 50 mM (950 ppm), is commonly used as a serine/threonine phosphatase inhibitor during cell lysis. Therefore, it is possible that F^−^ directly inhibits the alkaline phosphatase activity of SEAP without causing an ER stress-mediated decrease in SEAP secretion. To address this issue, we collected cell-free medium containing SEAP from LS8-SEAP cells grown in the absence of F^−^. Recombinant SEAP thus obtained was then incubated with different doses of F^−^ for 6 and 24 hr. As shown in Figure 2, no significant decrease in SEAP activity was observed (*p* > 0.05) until the F^−^concentration reached 50–100 mM. Thus, the decrease in the SEAP activity observed in our experiments can be attributed to reduced SEAP secretion and not to direct inhibition of phosphatase activity by F^−^.

### Intracellular accumulation of SEAP and activation of the UPR

**Figure 3:**
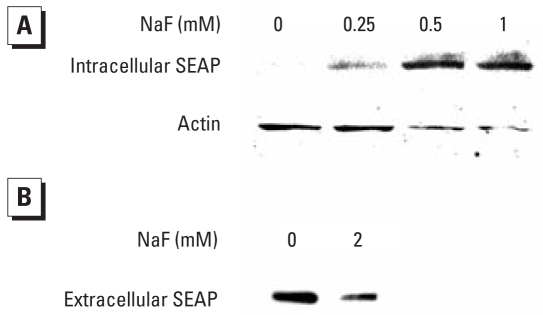
Immunoblots showing the effect of F^−^treatment on intracellular SEAP accumulation in LS8-SEAP cells treated with NaF for 24 hr. (*A*) Cell lysates. (*B*) Medium supernatants. Note the increase in intracellular SEAP (*A*) and a corresponding decrease in extracellular SEAP (*B*), suggesting protein retention within the cell. Blots were stripped and probed for actin as a loading control.

**Figure 4:**
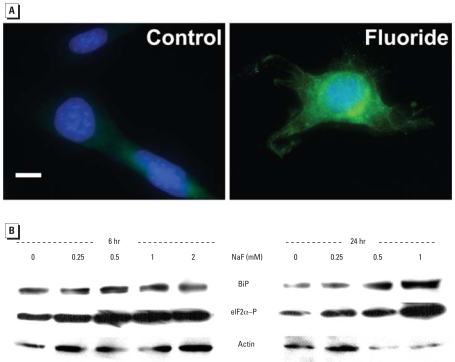
Effect of F^−^ on activation or induction of the UPR markers PERK, eIF2α, and BiP. (*A*) PERK phosphorylation in LS8-SEAP cells treated with 0.125 mM (2.4 ppm) NaF for 18 hr. PERK is phosphorylated (green) in NaF-treated cells but not in control cells; nuclei were stained with DAPI (blue). Bar = 10 μm. (*B*) Immunoblots probed for BiP and for the phosphorylated form of eIF2α(eIF2α–P) in LS8-SEAP cells treated with NaF for 6 or 24 hr. Blots were stripped and probed for actin as a loading control.

Any factor that disturbs ER homeostasis could lead to the intra-cellular accumulation of misfolded or unfolded proteins, causing ER stress. As shown in Figure 3A, treatment of LS8-SEAP cells with F^−^ results in a dose-dependent increase in the intracellular accumulation of SEAP protein. Conversely, secretion of SEAP into the medium decreases (Figure 3B). These immunoblot results indicate that F^−^ interferes with the secretion of SEAP and, presumably, other endogenous secretory proteins. We also demonstrate a concurrent induction of the ER stress-induced UPR signaling pathway along with the observed decrease in SEAP secretion (Figure 4). The UPR proximal sensor, PERK, is a transmembrane serine/threonine kinase activated by autotransphosphorylation (Harding et al. 2000). Activated PERK phosphorylates eIF2α (reviewed by Kimball 1999). This leads to transient translation attenuation, allowing the cells to cope with the proteins that have already accumulated in the ER. As shown in Figure 4A, PERK is activated (phosphorylated) in LS8-SEAP cells treated with 0.125 mM (2.4 ppm) F^−^. Immunoblots show translational induction of the molecular chaperone BiP, as well as phosphorylation of the PERK target eIF2α (Figure 4B) within 6 hr of F^−^ treatment. BiP remains induced after 24 hr of treatment, especially in cells treated with higher doses of F^−^ (≥19 ppm).

**Figure 5:**
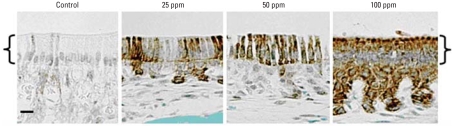
Effect of F^−^ on phosphorylation of eIF2αin maturation stage ameloblasts in mice given drinking water containing either 0, 25, 50, or 100 ppm F^−^ *ad libitum.* Immunohistochemistry was performed on incisor sections with antiserum specific for phosphorylated eIF2α. Note that significant staining occurred in ameloblasts (denoted by brackets) in the 25-ppm treatment group and that this staining intensified and spread to the papillary layer (beneath brackets) in the 100-ppm treatment group. Significant staining was not observed in identically treated secretory stage ameloblasts (not shown). Bar = 25 μm.

We next asked if maturation stage ameloblasts from mice drinking F^−^-treated water (0, 25, 50, or 100 ppm in drinking water for approximately 3–4 weeks) initiated phosphorylation of eIF2α*in vivo*. As shown in Figure 5, eIF2αwas phosphorylated at the lowest dose tested (25 ppm). The amount of eIF2αphosphorylation *in vivo* correlated positively with F^−^ dose, suggesting an increase in the magnitude of ER stress with increasing doses of F^−^.

## Discussion

Fluorosed enamel is characterized by hypomineralization, increased protein content, and greater surface and subsurface porosity. The most significant characteristic of fluorosed enamel is its increased protein content (Holland 1979a). Several studies have pointed toward F^−^-mediated inhibition of protein secretion and/or synthesis (Conconi et al. 1966; Godchaux and Atwood 1976; Helgeland 1976; Holland 1979a, 1979b; Lin et al. 1966; Vesco and Colombo 1970; Zhou et al. 1996). For example, F^−^ in the drinking water of rats inhibited protein removal from early maturation-stage incisor enamel (DenBesten 1986; DenBesten and Thariani 1992; Zhou et al. 1996). F^−^ has also been shown to inhibit insulin secretion in rats (Menoyo et al. 2005; Rigalli et al. 1990). However, a direct mechanism for F^−^-induced inhibition of protein secretion remains to be elucidated.

In this article, we show that NaF decreases secreted SEAP activity in a dose-dependent manner (Figure 1A). The effect is mediated only by NaF and not by NaCl (Figure 1B), suggesting that F^−^ is responsible for the observed decrease in protein secretion. We also demonstrate that the low concentrations of F^−^ used in our experiments do not directly inhibit recombinant SEAP activity (Figure 2). Thus, F^−^ does not interfere with our assay system.

Accumulation of excess protein within the ER is a hallmark of ER stress. We found that with an increase in F^−^ dose, increasing quantities of SEAP accumulate intracellularly (Figure 3). Thus, the observed decrease in SEAP secretion is at least partially due to ER stress-mediated protein retention within the cells. Our results with F^−^ are similar to reports using the well-characterized ER-stress inducer thapsigargin. SEAP also accumulated in the ER after thapsigargin treatment (Hiramatsu et al. 2006a).

F^−^ induces ER stress and initiates the UPR, as demonstrated by the induction of the molecular chaperone BiP and by phosphorylation of PERK and eIF2α ^(^Figure 4). We also demonstrated phosphorylation of eIF2α*in vivo* in ameloblasts of mice treated *ad libitum* with F^−^ at doses of ≥ 25 ppm (Figure 5). It must be noted that a higher F^−^ dose is required to cause flourosis in a mouse (25 ppm) compared with a human (~ 2 ppm). This may be because the continuously erupting mouse incisor ameloblast progresses from the secretory stage to the final maturation stage in a matter of weeks, whereas this progression occurs over several years for human teeth. Thus, human ameloblasts have a much longer exposure to F^−^ present in drinking water than do mouse ameloblasts. Second, rodents do appear to more efficiently clear F^−^from their bodies compared with humans (Angmar-Mansson and Whitford 1984). The immunostaining for phosphorylated eIF2αobserved in maturation stage ameloblasts exposed to 25 ppm F^−^ is highly significant, because 25 ppm F^−^ is the threshold concentration where F^−^-susceptible mice will have fluorosis (Everett et al. 2002).

F^−^-induced ER stress and subsequent inhibition of protein secretion is consistent with prior *in vivo* studies demonstrating F^−^-mediated disruption in the export of proteins from the ER (Kruger 1968; Matsuo et al. 1996, 2000). Furthermore, 100 ppm Fin rat drinking water delays by as much as 30% the modulation cycle of the apical ends of ameloblasts between a ruffle-ended and smooth-ended morphology during the maturation stage of enamel development (Smith et al. 1993). This modulation is thought to assist the ameloblasts in removing H^+^ ions from the enamel matrix, and its inhibition by F^−^ is consistent with a decrease in the translation of proteins required for the modulation to occur. Taken together, these observations support our results suggesting that F^−^ causes ER stress in ameloblasts and induces the UPR, which initiates eIF2α phosphorylation with subsequent attenuation of protein synthesis and secretion.
